# Improving the diagnosis of meningitis due to enterovirus and herpes simplex virus I and II in a tertiary care hospital

**DOI:** 10.1186/1471-2334-13-487

**Published:** 2013-10-21

**Authors:** Eduardo Casaroto, Alexandre R Marra, Fernando Morgadinho S Coelho, Joao Renato Rebello Pinho, Roberta Sitnik, Fernando Colombari, Elivane Silva Victor, Nair Hideko Muto, Carlos Senne, Oscar Fernando Pavão dos Santos, Michael B Edmond

**Affiliations:** 1 Intensive Care Unit, Hospital Israelita Albert Einstein, São Paulo, Brazil; 2 Instituto Israelita de Ensino e Pesquisa Albert Einstein, Hospital Israelita Albert Einstein, São Paulo, Brazil; 3 Division of Medical Practice, Hospital Israelita Albert Einstein, São Paulo, Brazil; 4 Laboratory of Molecular Biology, Hospital Israelita Albert Einstein, São Paulo, Brazil; 5 Department of Internal Medicine, Virginia Commonwealth University School of Medicine, Richmond, Virginia, USA

## Abstract

**Background:**

Enterovirus and herpes simplex viruses are common causes of lymphocytic meningitis. The purpose of this study was to analyse the impact of the use molecular testing for Enteroviruses and Herpes simplex viruses I and II in all suspected cases of viral meningitis.

**Methods:**

From November 18, 2008 to November 17, 2009 (phase II, intervention), all patients admitted with suspected viral meningitis (with pleocytosis) had a CSF sample tested using a nucleic acid amplification test (NAAT). Data collected during this period were compared to those from the previous one-year period, i.e. November 18, 2007 to November 17, 2008 (phase I, observational), when such tests were available but not routinely used.

**Results:**

In total, 2,536 CSF samples were assessed, of which 1,264 were from phase I, and 1,272 from phase II. Of this total, a NAAT for Enterovirus was ordered in 123 cases during phase I (9.7% of the total phase I sample) and in 221 cases in phase II (17.4% of the total phase II sample). From these, Enterovirus was confirmed in 35 (28.5%, 35/123) patients during phase I and 71 (32.1%, 71/221) patients during phase II (p = 0.107). The rate of diagnosis of meningitis by HSV I and II did not differ between the groups (13 patients, 6.5% in phase I and 13, 4.7% in phase II) (p = 1.0), from 200 cases in phase I and 274 cases in phase II.

**Conclusions:**

The number of cases diagnosed with enteroviral meningitis increased during the course of this study, leading us to believe that the strategy of performing NAAT for Enterovirus on every CSF sample with pleocytosis is fully justified.

## Background

Acute meningitis is a frequent syndrome encountered in emergency rooms. Enteroviruses (EV) accounts for 80% to 85% of all cases and are the most common pathogens associated with this disease, but bacterial agents such as *Neisseria meningitidis, Streptococcus pneumoniae and Haemophilus influenzae* are also common agents associated with this clinical picture. It is very important to achieve a rapid etiologic diagnosis to differentiate viral and bacterial meningitis so as to guide the introduction of antibiotic therapy early or avoid its unnecessary use for viral diseases. Among EV, Coxsackie and Echovirus are the predominant serotypes, infecting young adults and children primarily [1-5].

Other viruses may be associated with lymphomonocytic meningitis, such as the mumps virus (*Paramyxoviridae*). In adults, arboviruses may also be found, occurring in specific geographic regions, often as epidemic outbreaks (*Saint Louis encephalitis*, La Crosse virus, Jamestown Canyon, etc.) [6].

Although they account for only 0.5% to 3% of all cases of lymphomonocytic meningitis, meningitis and/or meningoencephalitis caused by Herpes simplex (HSV) I and II are even more important owing to their potential clinical severity in the case of meningoencephalitis, and the potential for therapeutic intervention [1].

The preferred diagnostic test to identify the etiology of viral meningitis, especially by EV and HSV 1/2, is the nucleic acid amplification test or NAAT. The advantages of this test, whether using polymerase chain reaction (PCR) or nucleic acid sequence-based amplification (NASBA), are its high sensitivity, quick results and potential to determine the need for antiviral treatment [1,4,7].

In most cases, the clinician must differentiate between viral and bacterial meningitis because the high mortality rates and serious sequelae associated with acute bacterial meningitis require the start of antimicrobial treatment before culture results are available. The antimicrobial treatment of bacterial meningitis may last 7 to 21 days. The need for resampling cerebrospinal fluid (CSF) depends primarily on the patient’s response [8].

The performance of a NAAT for EV and HSV 1/2 can help clinicians to determine the best approach in each case, avoiding unnecessary intravenous antibiotics, lumbar punctures, side effects from hospitalization, and may reduce the hospital stay of patients with meningitis, when a definite diagnosis of viral (and not bacterial) meningitis is made [1,7,9-11].

The purpose of the present study was to analyse the impact of the use molecular testing (NAAT) for EV and HSV 1/2 in all suspected cases of viral meningitis seen at the Hospital Israelita Albert Einstein (HIAE) and compare the results with a previous period when those tests were available but not requested routinely.

## Methods

### Study site and period

The study was conducted at Hospital Israelita Albert Einstein (HIAE) and its satellite units providing emergency care, in the city of São Paulo, in the time period from November 18, 2008 to November 17, 2009 (phase 2, intervention). At the start of the study CSF sample collection for Enterovirus and Herpes virus 1 and 2 NAAT was routinely implemented for all patients with suspected viral meningitis seen at the emergency units of HIAE.

We developed lectures (four education sessions during the study period) and a viral meningitis protocol for physicians, principally the infectious diseases physicians, neurologists, pediatricians and emergency medicine doctors. This educational program was reinforced through the Continuing Medical Education (CME) Office. Per the protocol, all CSF samples were tested for cell count, protein, glucose, Gram stain, and bacterial culture. All CSF samples with ≥4 WBCs/mm^3^ were automatically tested for EV and HSV 1/2 via NAAT. Data collected in this period were compared to those from the one-year period before this routine was implemented, i.e. November 18, 2007 to November 17, 2008 (phase 1, observational). In phase 1, the patient’ physician decided which patient might have viral meningitis, and ordered the EV and HSV 1/2 NAATs on a case by case basis.

In phase 2, all patients admitted with suspected viral meningitis were included, irrespective of their age. Patients were excluded from the study if they had normal CSF cellularity (less than 4 cells/mm3) and clinical chemistry parameters, such as glucose, protein, chloride and lactate, which ruled out meningitis, or if they had bacterial meningitis documented by a positive gram stain or latex test.

The case report form included epidemiologic data (gender, age, suspected diagnosis, number of lumbar puncture attempts), clinical data (signs and symptoms, such as headache, nausea and/or vomiting, neck stiffness, temperature, photophobia), prior use of medications (corticosteroids, immunosuppressive drugs, antimicrobials), laboratory data (hemoglobin and hematocrit, WBC, platelet count, sodium, potassium, creatinine, BUN, blood glucose, alkaline phosphatase, C-reactive protein, CSF analysis, detection of Enterovirus and Herpes virus by NAAT), and date of hospital discharge .

### Methodology

The institution’s molecular biology laboratory used the NASBA (Nucleic Acid Sequence Based Amplification) methodology. The detection of Enterovirus and Herpes simplex 1 and 2 was performed with Nuclisens EasyQ kits (Biomérieux, Marcy-l′Etoile, France) specific for these pathogens, following the manufacturer’s instructions. A 1-mL sample of CSF was obtained from each patient. Nucleic acids were extracted using the EasyMag equipment and then submitted to amplification using the NucliSENS EasyQ® HSV 1/2 and NucliSENS EasyQ® Enterovirus kits. The HSV 1/2 kit has probes labeled with different fluorophores, which identify and differentiate HSV1 from HSV2. The kit for Enterovirus, has probes that detect preserved regions across the different serotypes of Enterovirus, including Polio 1–3, Coxsackie A2-12, A15-18, A20, A21, A24, Coxsackie B1-6, Echovirus 1–9, 11–15, 17–21, 24–27, 29–33, and Enterovirus 68–71. In terms of analytic sensitivity, the 50% and 95% exhaustive coefficients have been determined to be 93 and 263 copies per nucleic acid extract, respectively. This means that a sample at concentrations of 93 and 263 copies per nucleic extract will be detected with a positivity rate of 50 and 95% respectively when exhaustively tested. Both kits also have a probe to detect an internal control, monitoring the quality of the process from the extraction phase.

During phase I, molecular analyses were performed only 3 days a week, and the turnaround time was approximately 24 hours. In phase II, NAAT for EV and HSV 1/2 was available 7 days per week, 24-hours per day and the target time to release the result was 12 hours.

### Statistical analysis

Continuous variables were compared using the Student’s t test if they showed a normal distribution, and the Mann–Whitney test if they did not. Differences in proportions were compared using the Pearson’s chi-square test or the Fisher exact test, as appropriate. Mean values were reported ± 1 standard deviation. The p value was defined as 0.05 and all tests with statistical significance were two-tailed. All statistical analyses were developed using the Statistical Package for the Social Sciences software (SPSS, Chicago, IL, USA,11.5).

### Informed consent exemption

The present study was approved by the Ethics Committee of HIAE. Considering that the NAAT is routinely performed in the hospital, and that the study included only the recording of laboratory data and a search in medical records, our institutional review board waived the need for written informed consent from the participants.

## Results

In total, 2,536 CSF samples were assessed, of which 1,264 were from the pre-study period (phase I) and 1,272 from the post-study period (phase II). Of this total, a NAAT for EV was ordered in 123 cases during phase I (9.7% of the total phase I sample) and in 221 cases in phase II (17.4% of the total phase II sample). From these, EV was confirmed in 35 patients during phase I and 71 patients during phase II (p = 0.107). The diagnosis of meningitis by HSV 1 and 2 did not differ between the groups (13 patients in phase I and 13 in phase II) (p = 1.0), from 200 cases in phase I and 274 cases in phase II (Table 1).

**Table 1:** Results of nucleic acid amplification testing (NAAT)

**Variables**	**Phase 1**	**Phase 2**	**Total**
Lumbar punctures performed	1,264	1,272	2,536
NAAT performed for Enterovirus	123	221	344
NAAT performed for Herpes virus 1 and 2	200	274	474
Viral meningitis cases ( Enterovirus + Herpes virus 1 and 2)	48	84	132
Meningitis by Enterovirus – n (%)*	35 (73)	71 (84)	106 (80)
Meningitis by Herpes virus 1 and 2 - n (%)**	13 (27)	13 (16)	26 (20)

*p-value = 0.107.

**p-value = 1.0.

Among those patients with viral meningitis (either by EV or by HSV 1/2- see Table 2), 52.1% in phase I and 36.9% in phase II were females (p = 0.9). The mean age of the patients was 20.0 years (SD 16.4) in phase I and 17.5 years (SD 17.3) in phase II (p = 0.43). The number of specimens by age was for EV: 0–5 years (46.2%, 49/106), 6–17 years (24.5%, 26/106), 18+ years (29.3%, 31/106); and for HSV1/2: 0–5 years (no cases), 6–17 years (3.8%, 1/26 --one case for HSV 1), 18+ years (96.2%, 25/26 – 25 cases for HSV 2). In phase I 23.4% and in phase II 14.3% had some comorbidity (p = 0.19). The mean number of days of hospitalization was 2.9 (SD 2.6) in phase I and 2.7 (SD 3.9) in phase II (p = 0.69).

**Table 2:** Sample including Enterovirus and HSV 1/2

		**Phase 1**		**Phase 2**		**P value**
		**n**	**%**	**n**	**%**	
Gender	Female	25	52.1	31	36.9	0.09
	Male	23	47.9	53	63.1	
Meningitis	Herpes	13	27.1	13	15.5	0.10
	Enterovirus	35	72.9	71	84.5	
Presence of any comorbidity		11	22.9	12	14.5	0.19
Headache		42	87.5	75	92.6	0.99
Nausea and vomiting		30	62.5	57	70.4	0.66
Fever (T > 37.7°C)		35	72.9	55	67.9	0.24
Stiff neck		19	39.6	40	49.4	0.44
Predominant cellularity in 1st CSF sample	Neutrophilic	11	22.9	29	34.9	0.15
	Lymphomonocytic	37	77.1	54	65.1	
Result of NAAT for Enterovirus	Negative	12	25.5	13	15.5	0.02
	Positive	35	74.5	71	84.5	
Result of NAAT for Herpes simplex	Negative	29	60.4	64	76.2	0.18
	Positive for HSV type I	1	2.1	0	0	
	Positive for HSV type II	12	25	13	15.5	
Predominant cellularity in 2nd CSF sample	Neutrophilic	0	0	0	0	***
	Lymphomonocytic	7	100	7	100	
Use of antibacterials to treat meningitis		7	14.6	19	22.6	0.264
Underwent imaging exam (brain)		22	45.8	41	49.4	0.694
		**Phase 1**		**Phase 2**		**P value**
		**Mean**	**SD**	**Mean**	**SD**	
Age (years)		20.0	16.5	17.5	17.3	0.43
Length of hospital stay (days)		2.9	2.6	2.7	4.0	0.69
Time from symptom onset to admission		2.13	2.23	1.60	1.26	0.08
Leukocytes in 1st CSF sample (n/mm^3^)		262.9	259.6	205.2	217.7	0.17
Neutrophils in 1st CSF sample (%)		29.9	28.5	38.3	29.2	0.11
Lymphocytes in 1st CSF sample (%)		50.3	28.3	43.9	28.2	0.21

***testing not possible.

In the group of patients with viral meningitis due to EV or HSV 1/2 (Table 2), the most commonly found symptoms included: headache (93.3% in phase I and 92.6% in phase II) (p > 0.99), nausea and vomiting (66.7% vs. 70.4%) (p = 0.66), fever (77.8% vs. 67.9%) (p = 0.24) and neck stiffness (42.2% vs. 49.4%) (p = 0.44).

CT scans were performed on 45.8% of the patients in phase I and 49.4% of the patients in phase II (p = 0.69); in phase I, 14.6% received antibacterial treatment versus 22.6% in phase II (p = 0.26) (Table 2). However, the antibacterial treatment duration was less than 24 hours in 78.3% of the cases of meningitis due to EV.

Concerning CSF characteristics, in phase I, 77.1% showed predominantly lymphomonocytic cellularity at the first sampling versus 65.1% in phase II (p = 0.15). The WBC results showed a median of 162.5 and an interquartile range (IQR) of 85–350 in phase I versus 118 and 59.5-313.5 in phase II (p = 0.174) (Table 2). In about 10% of cases (in both phases), the number of leukocytes in the first CSF sample exceeded 500 cells per mm^3^. The glucose, protein and lactate levels in the CSF were quite similar in both groups. In none of them were bacteria detected by gram stain, latex agglutination, or a positive culture.

When we analyzed only the subgroup with meningitis by EV (Table 3), we found that the mean hospital stay was 2.4 days (SD 2.3) in phase 1 versus 1.9 days (SD 2.4) in phase 2 (P = 0.25). A lymphomonocytic pleocytosis in the CSF was 71.4% in phase 1 and 59.2% in phase 2 (P = 0.21). The number of patients treated with antibacterials was 6 (17.1%) in phase 1 versus 18 (25.4%) in phase 2 (P = 0.34). However, there was no record of the duration of antibiotics use, only whether or not they were used.

**Table 3:** Demographic data of patients with meningitis due to Enterovirus

		**Phase 1**		**Phase 2**		**P value**
		**N = 35**	**%**	**N = 71**	**%**	
Gender	Female	14	40	19	26.8	0.16
	Male	21	60	52	73.2	
Presence of any comorbidity		8	23.5	5	7	0.02
Headache		29	90.6	63	92.6	0.70
Nausea and vomiting		30	66.7	57	70.4	0.66
Fever > 37.7°C		26	81.3	50	73.5	0.39
Stiff neck		13	40.6	32	47.1	0.54
Predominant cellularity in 1st CSF sample	Neutrophilic	10	28.6	29	40.8	0.21
	Lymphomonocytic	25	71.4	42	59.2	
Predominant cellularity in 2nd CSF sample	Neutrophilic	0	0	0	0	***
	Lymphomonocytic	4	100	3	100	
Use of antibacterials to treat meningitis		6	17.1	18	25.4	0.34
Underwent imaging exam (brain)		13	37.1	29	40.8	0.71
		**Phase 1**		**Phase 2**		**P value**
		**Mean**	**SD**	**Mean**	**SD**	
Age (years)		12.5	11.7	12.5	12.8	0.98
Length of hospital stay (days)		2.4	2.3	1.9	2.4	0.25
Time from symptom onset to admission		2.34	2.55	1.58	1.28	0.10
Leukocytes in 1st CSF sample (n/mm^3^)		254.9	262.5	184.5	208.5	0.13
Neutrophils in 1st CSF sample (%)		38.7	27.3	43.9	27.8	0.36
Lymphocytes in 1st CSF sample (%)		40.2	23.1	37.5	24.6	0.58

***testing not possible.

After phase II, we have continued to monitor the number of EV meningitis cases over the years, and the number of diagnosed cases increased gradually reaching 30% (72/242) in 2009, 27% (71/260) in 2010, 46% (112/242) in 2011 and 48% (56/116) in the first half of 2012 (Table 4).

**Table 4:** Number of cases with positive result for Enteroviruses from January 2008 to June 2012

**Month**	**2008**			**2009**			**2010**			**2011**			**2012**		
	**Total**	**Positive**		**Total**	**Positive**		**Total**	**Positive**		**Total**	**Positive**		**Total**	**Positive**	
		**n**	**%**		**n**	**%**		**n**	**%**		**n**	**%**		**n**	**%**
January	10	6	60	8	1	13	16	5	31	14	3	21	6	1	17
February	16	3	19	13	4	31	13	3	23	15	7	47	12	5	42
March	18	5	28	16	5	31	16	2	13	26	15	58	18	9	50
April	3	1	33	21	8	38	21	7	33	26	15	58	35	24	69
May	15	2	13	22	9	41	23	6	26	24	12	50	23	11	48
June	15	3	20	24	7	29	26	3	12	15	10	67	22	6	27
July	5	1	20	24	7	29	29	8	28	18	4	22	-	-	-
August	8	2	25	17	3	18	18	6	33	16	4	25	-	-	-
September	5	1	20	10	4	40	22	9	41	15	7	47	-	-	-
October	10	5	50	27	5	19	25	11	44	26	15	58	-	-	-
November	25	11	44	21	6	29	27	5	19	17	7	41	-	-	-
December	13	6	46	39	13	33	24	6	25	30	13	43	-	-	-
Total	143	46	32	242	72	30	260	71	27	242	112	46	116	56	48

## Discussion

EV infections are common in children and adults, even though children are more susceptible to infection [1,12]. They may cause several diseases such as conjunctivitis, upper and lower respiratory tract infections, meningitis, encephalitis, encephalomyelitis and acute flaccid paralysis [3,7,13].

Enteroviruses belong to the *Picornaviridae* family, consist of a single strand of RNA [13], and include Coxsackie viruses A and B, Echovirus, and Poliovirus, among others, totaling more than 80 serotypes [1-3,13]. Some of these serotypes are more closely associated with meningitis than others [13].

EV Transmission occurs mainly through the fecal – oral route, but the oral – oral and the respiratory routes are also possible [2,14]. The most common form of CNS infection is through viremia, with some serotypes exhibiting more marked neurotropism or neurovirulence [2].

Approximately 80 to 90% of all cases of aseptic meningitis, in which a pathogen is identified, are enteroviruses. [1,2,4,7,10,11,13] When we analyze the adult population (>16 years of age), EV infection appears in about 40% of the cases of aseptic meningitis followed by HSV-2 and VZV (17% each), EBV (12%), HSV-1, and mumps virus (7% each) [15]. The varicella-zoster virus seems to be underestimated as a cause of aseptic meningitis and encephalitis [5].

In Brazil, Santos et al. have shown that Echovirus 30 was the most frequently agent isolated from CSF samples of patients with viral meningitis [14].

In most enteroviral infections, the incubation period ranges from 3 to 10 days [2], and several viruses causing meningitis have a seasonal distribution, with a predominance in summer and autumn [11,16], a pattern we have not observed in our sample.

Symptoms vary depending on the affected age range and include headache, fever, irritability, stiff neck, photophobia, nausea and vomiting [1-3,11,13,14].

The most commonly used diagnostic methods include CSF analysis, culture, serology, and nucleic acid amplification [1,3]. Establishing a definite diagnosis is important to reduce the hospitalization period and the use of antibiotics [1,17] and these are reasons favoring the diagnosis of viral meningitis by molecular methods (NAAT).

The CSF analysis in viral meningitis usually reveals predominantly mono-morphonuclear pleocytosis, more often between 100 and 300 leukocytes/mm^3^, normal or slightly elevated protein, and normal glucose [1,2]. However, there seems to be different CSF patterns depending on the pathogen involved [15]. The CSF may show up to 1,000 leukocytes per mm^3^, mainly polymorphonuclear cells, in the earlier phases of the disease, making diagnosis even more difficult [1,2,7,10].

Graham and Murdoch have shown that more than half of the patients with pleocytic CSF had a polymorphonuclear pattern, despite a confirmed viral etiology [17].

However, the diagnosis of EV meningitis is often made in the absence of pleocytosis or increased levels of protein, especially in children [1,2,9,17].

Culture is the most traditional method [9], but EV take from 4 to 8 days to grow in cell culture, therefore the culture can seldom contribute to the patient’s management [1,11]; its use, therefore, is no longer recommended and has been abandoned in routine laboratory practice [18]. In addition, it has low sensitivity and must be performed by highly qualified personnel [7,11]. Many patients with meningitis by EV are hospitalized and treated with parenteral antibiotics until they show clinical improvement and negative cultures after 48 hours of incubation [7].

Serological methods are currently used to confirm infection during outbreaks caused by a single serotype of EV [1]. A minimum 4-fold increase in antibody titers is necessary between the acute and convalescent phases [2]. The quantitative interpretation of a single serum sample is of no value, since there is great variation between the titers for different serotypes in healthy subjects. Considering that there are more than 60 antigenically different serotypes without shared antigens, serological techniques are not very practical [2].

On the other hand, nucleic acid amplification techniques allow for a quick diagnosis of enteroviral infections [1,7,9] and represent the best diagnostic method available at present [7,10,12]. Different commercial kits are currently available; they require a small sample volume, show higher sensitivity than viral culture, have 100% specificity, [1,2,4,18] and their cost is similar to that of a culture [7]. Over the study period, our laboratory worked with the only molecular technique for EV available on the market, the Nuclisens system for specific amplification of viral RNA using the NASBA methodology [19]. Since NAAT shows positivity well before viral cultures, it allows the reduction of unnecessary hospitalization as well as diagnostic and therapeutic interventions, especially in documented cases of enteroviral meningitis [1,4,7,10,11].

However, in many centers, NAAT is not available on a daily basis to the point that some patients admitted during weekdays remained in hospital one day less and used less antibiotics when compared to those admitted during the weekend [10]. At the beginning of the present study, molecular analyses were performed only 3 days a week, at the approximate cost of $175, and the turnaround time was approximately 24 hours. At present, PCR for enterovirus is available at our hospital 7 days a week, 24-hour a day and the target time to release the result is 12 hours, at an approximate cost of $85. This has increased the routine use of this technology.

Studies show that whenever NAAT results for Enterovirus are available, hospitalization can be reduced by at least 12 to 24 hours, which in turn significantly reduces the costs. Other previously reported benefits include a shorter stay in inpatient care units, fewer requests for computerized tomography, magnetic resonance imaging, and electroencephalograms, as well as shorter-term antibiotic therapies [1,9,10]. Our data failed to show these same results, however this may have been due to inadequate sample size. Unnecessary antibiotic use and the corresponding increase in bacterial resistance rates are topics of interest in the approach to EV meningitis [1].

The ability to detect and quickly differentiate EV-associated diseases from those related to other viruses and/or bacteria represents a critical target from the therapeutic, prognostic and epidemiologic points of view [14].

Since 2008, when our hospital started to implement NAAT for EV, the number of diagnosed cases increased gradually reaching 112 positive results from 242 suspect cases (46.3% of the sample) in 2011 (Table 4). The number of diagnosed cases practically doubled, while the same was not observed with HSV, highlighting the benefits of using NAAT on a routine basis. It is also worth mentioning that our study failed to show any reduction in hospitalization time or exposure to antimicrobial therapy, since our sample included only those patients with a documented diagnosis of viral meningitis. During phase 1, it is possible that patients with enteroviral meningitis not promptly diagnosed by the usual methods received antibiotics for a few days, have undergone repeat spinal tap and have remained in hospital for longer than necessary, simply due to the absence of a documented viral diagnosis.

We are aware of the fact that, at present, there is no effective antiviral therapy for EV, and management is limited to symptomatic measures [2,4,11,16]. Most cases resolve spontaneously, [11,16] usually in less than 7 days [1], but the molecular diagnosis of meningitis by EV and HSV 1/2 is beneficial in many ways. It reduces the use of antibacterials and their associated risks, shortens hospitalization, allows for fewer lumbar punctures, helps reduce parental anxiety about the diagnosis and the likelihood of a bacterial infection and, saves costs for the hospital and the family.

This study had several limitations. This study was a protocol for improving viral meningitis diagnosis in our institution. It was performed at a single medical center, and thus our findings might not be generalizable to other hospitals. We did not create a prompt during the interventional phase (phase II) for clinicians to order both tests (EV and HSV). Pediatricians generally asked only EV and not HSV for children. We had also patients with meningoencephalitis or with encephalitis for whom EV was not performed. This is one of the reasons for the differences between the number of EV and HSV 1/2 tests ordered. One other limitation was that we may have missed cases because we required pleocytosis for testing for EV or HSV 1/2.

The mere fact that the number of cases diagnosed with EV meningitis increased by almost 100% during the implementation of this protocol leads us to believe that the strategy of performing NAAT for EV on every CSF sample is extremely important.

## Conclusion

Based on this study, our hospital decided to implement the strategy and also to use it as a quality indicator.

## Competing interests

The authors have declared that no competing interests exist.

## Authors’ contributions

EC, RS, CS, ESV, NHM and FC participated in the data collected and data analysis. ARM, FMSC, OFPS and MBE participated in the design and coordination. EC, ARM, FMSC, JRRP, RS, FC, NHM, OFPS and MBE helped to draft the manuscript and to provide critical review of the manuscript. All authors read and approved the final manuscript.

## Pre-publication history

The pre-publication history for this paper can be accessed here:

http://www.biomedcentral.com/1471-2334/13/487/prepub
